# Using the optokinetic response to study visual function of zebrafish

**DOI:** 10.3791/1742

**Published:** 2010-02-02

**Authors:** Su-Qi Zou, Wu Yin, Ming-Jing Zhang, Chun-Rui Hu, Yu-Bin Huang, Bing Hu

**Affiliations:** Laboratory of Neurodevelopment and Repair, Department of Neurobiology and Biophysics, School of Life, University of Science and Technology of China

## Abstract

Optokinetic response (OKR) is a behavior that an animal vibrates its eyes to follow a rotating grating around it. It has been widely used to assess the visual functions of larval zebrafish^1-5^. Nevertheless, the standard protocol for larval fish is not yet readily applicable in adult zabrafish. Here, we introduce how to measure the OKR of adult zebrafish with our simple custom-built apparatus using a new protocol which is established in our lab. Both our apparatus and step-by-step procedure of OKR in adult zebrafish are illustrated in this video. In addition, the measurements of the larval OKR, as well as the optomotor response (OMR) test of adult zebrafish, are also demonstrated in this video. This OKR assay of adult zebrafish in our experiment may last for up to 4 hours. Such OKR test applied in adult fish will benefit to visual function investigation more efficiently when the adult fish vision system is manipulated.

Su-Qi Zou and Wu Yin contributed equally to this paper.

**Figure:**
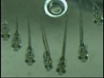


## Protocol

### Part I: Construct the OKR apparatus

Details are shown in video demonstration.

### Part II: OKR of Adult Zebrafish

If your lab has constructed an OKR apparatus, the key of adult zebrafish OKR is to hold the fish. In this part, we will show you step by step.

Our apparatus composes following parts similar to others mentioned. The circular fish chamber is 7.0 cm in outer diameter, 6.5 cm in inner diameter and 7.0 cm in high. The drum around fish chamber is 8.5 cm in out diameter, 7.5 cm in inner diameter and 7.0 cm in high. It could rotate clockwise and counter-clockwise with the speed from 20~70 rotations per min (rpm). Eleven cycles of black and white stripes are drawn on a tracing paper which adhered on the outside of the drum. The light system is composed of two circle light tubes in 21 cm diameter.


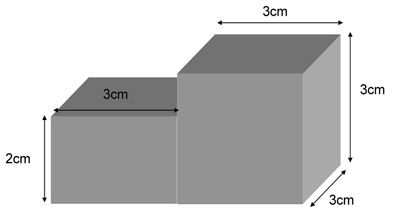
 **Figure 1. The model of sponge.** The cliff made in this middle is to make sure that the visual field was not being sheltered.

A sponge as shown in Fig.1 composed by two parts. The left part is length 3 cm x width 3 cm x high 2 cm. The right part is length 3 cm x width 3 cm x high 3 cm.
Put this sponge in the bottom of fish chamber and pour rearing water in about 2 cm depth. Air bubble which hide in the sponge should be driven out.
Insert a pin in the front of right part of sponge, about 2 mm behind the cliff.
Anesthetize an adult zebrafish in MS-222.
Put the zebrafish on the sponge with dorsal up and allow one side of its pectoral fin before the first pin.
The second pin is inserted behind the other pectoral fin. It could avoid zebrafish shrinking body by cooperating with the first pin (also see in Fig. 2).
Clamp its tail with an artery clamp and fix the artery clamp in a piece of rubber (modeling clay) which is adhered on the fish chamber.
The third and fourth pin is planted in the site near cloacal pore of fish body. It could prevent zebrafish to wave its tail intensely.
The fifth pin is sited between the first and the third pin and then the sixth pin is on the other side of it. They could prevent zebrafish from shaking its body.
The seventh and eighth pin is inserted in the sites near the gill to keep the head orientation.
Pour rearing water in the fish chamber.
After the zebrafish wakes up, let it adapt for 5~10 minutes.
Switch on the light of this apparatus and let zebrafish adapt this lighting for 2 minutes.
Switch on the motor to drive the grating and allow zebrafish to adapt for 1 minute.
Turn on the camera and record the movement of eyes.
Analyze the eye angles^6^ and saccade numbers on your personal computer (A representative example of the eye angles analysis is shown in Fig.2).

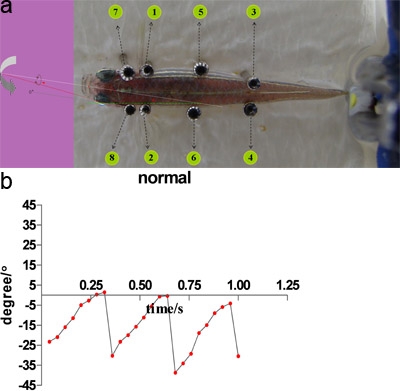
 **Figure 2.** The angle between eye axis and body axis. a)When the direction of grating is nasal-to-temporal, we defined the angle at the ahead of fish body is a negative angle and the angle at the fish body is a positive angle, vice versa (for brief review also see Ref. 6). Numbers in this figure represent the sequence of pins. b)OKR analysis example from one fish in normal group. An one-second film was transferred to a series frames and the eye angles of each frame was measured with Image-pro Plus software. Each point in this figure represents each frame we measured.

Notes:

All the pins are just used to prevent zebrafish from shaking its body, too tight will be harmful to fish.
Artery clamp could damage the tail as its contact area is small. Once the tail is free, it s very difficult to fix this fish. Wrap a bandage around the tail is recommended in some condition.
The seventh and eighth pin which are planted in the site near gill should not be shelter from their sight of grating.

### Part III. OKR of Larval Zebrafish

#### 1. Methylcellulose

Larval zebrafish can be immobilized in 6% methylcellulose solution while keep alive for a long time. Prepare methylcellulose solution as follows.

Boil 200 ml ddH_2_O in microwave oven about 3 minutes at high level.
Transfer the hot water to a stirring hotplate and stir it vigorously.
Add 12 g methylcellulose into the hot water gradually.
Keep stirring for 3 minutes after methylcellulose is well-dispersed.
Cool the solutions with ice water until it becomes clear, and then place it at 4°C.

#### 2. Larval Zebrafish

Pairs two male and a female of adult zebrafish in a mating tank at 17:00 after feeding. Next morning transfer them to a fresh mating tank and collect embryos after 1 hour.
The eggs are maintained in embryo medium in a 28.5±0.5°C incubator with the light: dark cycle of 14:10.
Eggs will be hatched out at 48 hours post fertilization (hpf) and the OKR will be tested at 72 hpf or later on. In this video, the larvaes are at 5 dpf.

#### 3. Procedure of OKR

Put the apparatus under the stereoscope equipped with CCD camera and switch on the light of this apparatus.
Add 20 ml 6%methycellulose in a 55-mm petri dish.
Place several larvaes in a row at centre of this dish.
Put an air bubble about a volume of 1 μl in the camera field, it could be as a mirror to reflect the direction of the grating.
Switch on the motor and record the movement of their eyes.

### Part IV. OMR of Adult Zebrafish

Adult zebrafish OMR could also be conducted with this apparatus, just place a column in the fish chamber for preventing fish swimming across centre (showed in Fig.3a, rotating velocity is 36 rpm, black-white cycles are 11).

Put a fish in this fish chamber.
Stand a column of 2 cm diameter in the centre of this fish chamber.
Adapt for one minute both in still grating and rotation grating.
Record the movement of zebrafish.
Analyze the coincidence rate of time on your personal computer (an example is shown in Fig.3b, rotating velocity is 36 rpm, black-white cycle is 11)

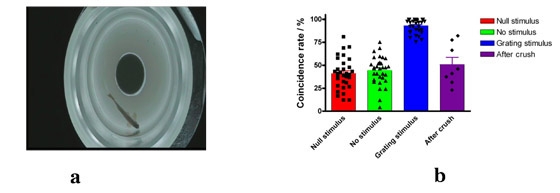
 **Figure 3. OMR of Adult Zebrafish**. (a) A white plastics column was placed at the centre of fish chamber for preventing fish swimming across it. (b) The coincidence rate  of adult zebrafish following the grating. The coincidence rate was defined as the proportion of the following grating time to the total recording time. The value of 50% indicates that the fish is swimming randomly while 100% indicates the fish following grating very well. Both optic nerves were crushed with a jewelry forceps about 10 seconds. The behavior of OMR appeared as a random swimming after crush.

## Discussion

According to the definition of OKR, it is a phenomenon that animals move their eyes to pursue a rotating grating spontaneously. If this behavior has been conducted for a long time (e.g. above 6 hours), the eye muscles may be tired and the OKR will be attenuated.

Adult zebrafish OMR has been described completely by others^7^. In this video, we just show you this behavior with our apparatus. Once the optic nerves are crushed, they could not follow this grating very well.

We have performed OKR behavior test in adult, larval zebrafish and OMR in adults with our simple custom-built apparatus to evaluate the visual function of zebrafish. This OKR assay of adult zebrafish in our experiment could keep strong enough within a period of 4 hours without to harm it. This may open up OKR test in adult fish function study more efficiently.
